# Are computed tomography 3D measurements of the upper airways in mouth-breathing children in agreement with the ENT clinical diagnosis of obstruction?^☆^

**DOI:** 10.1016/j.bjorl.2018.01.006

**Published:** 2018-03-11

**Authors:** Bruno César Ladeira Vidigal, Carolina Morsani Mordente, Paula Loureiro Cheib, Flávio Ricardo Manzi, Letícia Paiva Franco, Helena Maria Gonçalves Becker, Bernardo Quiroga Souki

**Affiliations:** a Pontifícia Universidade Católica de Minas Gerais (PUC-Minas), Programa de Pós-Graduação em Odontologia, Belo Horizonte, MG, Brazil; b Pontifícia Universidade Católica de Minas Gerais (PUC-Minas), Odontologia, Belo Horizonte, MG, Brazil; c Universidade Federal de Minas Gerais (UFMG), Hospital das Clínicas, Ambulatório de Respirador Oral, Belo Horizonte, MG, Brazil; d Universidade Federal de Minas Gerais (UFMG), Faculdade de Medicina, Hospital das Clínicas, Ambulatório de Respirador Oral, Belo Horizonte, MG, Brazil

**Keywords:** Tomography, Mouth breathing, Nasal cavity, Oropharynx, Nasopharynx, Tomografia, Respiração bucal, Cavidade nasal, Orofaringe, Nasofaringe

## Abstract

**Introduction:**

Imaging studies have hystorically been used to support the clinical otorhinolaryngological evaluation of the upper respiratory tract for the diagnosis of obstructive causes of oral breathing.

**Objective:**

The objective of this study was to compare 3D volumetric measurements of nasal cavity, nasopharynx and oropharynx of obstructed mouth-breathing children with measurements of non-obstructed mouth-breathing children.

**Methods:**

This retrospective study included 25 mouth-breathing children aged 5–9 years evaluated by otorhinolaryngological clinical examination, flexible nasoendoscopy and full-head multi-slice computed tomography. Tomographic volumetric measurements and dichotomic otorhinolaryngological diagnosis (obstructed vs. non-obstructed) in three anatomical regions (the nasal cavity, nasopharynx and oropharynx) were compared and correlated. An independent sample *t*-test was used to assess the association between the 3D measurements of the upper airways and the otorhinolaryngological diagnosis of obstruction in the three anatomical regions. Inter- and intra-observer intraclass correlation coefficients were used to evaluate the reliability of the 3D measurements.

**Results:**

The intra-class correlation coefficients ranged from 0.97 to 0.99. An association was found between turbinate hypertrophy and nasal cavity volume reduction (*p* < 0.05) and between adenoid hyperplasia and nasopharynx volume reduction (*p* < 0.001). No association was found between palatine tonsil hyperplasia and oropharynx volume reduction.

**Conclusions:**

(1) The nasal cavity volume was reduced when hypertrophic turbinates were diagnosed; (2) the nasopharynx was reduced when adenoid hyperplasia was diagnosed; and (3) the oropharynx volume of mouth-breathing children with tonsil hyperplasia was similar to that of non-obstructed mouth-breathing children. The adoption of the actual anatomy of the various compartments of the upper airway is an improvement to the evaluation method.

## Resumo

**Introdução:**

O exame clínico otorrinolaringológico da via aérea superior tem sido historicamente feito com a ajuda de imagens radiográficas para diagnosticar causas obstrutivas da respiração bucal.

**Objetivo:**

O objetivo deste estudo foi comparar as medidas volumétricas em 3D da cavidade nasal, nasofaringe e orofaringe entre crianças com respiração bucal e obstrução respiratória, e crianças respiradoras bucais sem obstrução respiratória.

**Método:**

Estudo retrospectivo que inclui 25 crianças respiradoras bucais de 5 a 9 anos de idade. As crianças foram avaliadas por exame clínico otorrinolaringológico, nasofibroscopia flexível e tomografia computadorizada multi-slice. Medidas volumétricas obtidas tomograficamente de três regiões anatômicas (cavidade nasal, nasofaringe e orofaringe) foram correlacionadas e comparadas com diagnóstico dicotômico otorrinolaringológico (obstruído vs. não obstruído). Um teste *t* de amostra independente foi usado para avaliar a associação entre as medidas em 3D das vias aéreas superiores e o diagnóstico otorrinolaringológico de obstrução nas três regiões anatômicas. Os coeficientes de correlação intraclasse inter e intraobservador foram usados para avaliar a confiabilidade das medidas em 3D.

**Resultados:**

O coeficiente de correlação intraclasse variou de 0,97 a 0,99. Uma associação foi encontrada entre a hipertrofia de conchas e a redução do volume da cavidade nasal (*p* < 0,05) e entre a hiperplasia de tonsila faríngea e a redução do volume da nasofaringe (*p* < 0,001). Não foi encontrada associação entre a hiperplasia da tonsila palatina e a redução do volume da orofaringe.

**Conclusões:**

1) O volume da cavidade nasal estava reduzido nas crianças com diagnóstico de hipertrofia de conchas; 2) O volume da nasofaringe estava reduzido nas crianças com diagnóstico de hiperplasia de tonsila faríngea; e 3) O volume da orofaringe de crianças com respiração bucal e hiperplasia de tonsila palatina foi semelhante ao de crianças respiradoras bucais sem aumento da tonsila palatina. A adoção da mensuração anatômica dos vários compartimentos da via aérea superior complementa o método de avaliação.

## Introduction

An ENT clinical examination of the upper airway has been historically performed with the aid of radiographic images to diagnose obstructive causes of mouth breathing (MB).^1^ In recent decades, flexible nasoendoscopy has become a diagnostic tool.^2^ A clinical examination combined with flexible nasoendoscopy (FN) is the gold standard for the diagnosis of upper airway obstruction.^3^, ^4^ With the increasing use of computed tomography (CT) in several health science fields and the development of commercial software, new perspectives have occurred. This technology, among other features, enables faster and more reliable measurement of airway volume and area.^5^, ^6^, ^7^, ^8^, ^9^ CT images are reproducible, do not have magnification error, and allow 3D measurements. Volumetric airway measurements have been used in several studies and have been proposed as the gold standard scientific method for the study of breathing problems.^10^ However, validation of using 3D reconstructions for the diagnosis of obstructive tissues has not been presented.

Several obstructive factors are involved in the etiology of respiratory sleep disorders.^5^, ^11^, ^12^ Hypertrophic turbinates, adenoids and tonsils have an important effect, playing a major role in the etiology of respiratory obstruction of young patients.^6^, ^13^, ^14^ Early diagnosis of upper airway obstruction might contribute to a timely referral to ENT physicians, preventing complications and improving the quality of life of patients. Agreement between reduced CT volume measurements of the upper airways and an ENT diagnosis of obstruction could benefit scientific investigations, and eventually the clinical practice. Studies have shown that CT airway volume measurements, despite being highly reproducible within the same software, have low agreement when different programs and methodologies are used.^6^, ^8^, ^13^ A great variability in measurement values is found because the reference structures used for CT airway measurements^2^, ^3^, ^4^, ^8^, ^9^, ^11^, ^13^, ^15^ have been based on the criteria of individual researchers. The boundaries of the cavities that comprise the upper airways have not been based on anatomical landmarks; typically, they are based on the lines and planes that facilitate convenient reproducibility.

The purpose of this retrospective study was to investigate whether 3D volumetric measurements of the nasal cavity, nasopharynx and oropharynx of obstructed MB children are different from the measurements of MB children without the upper airway obstruction when precise anatomical landmarks boundaries are employed.

## Methods

### Sample

The Institutional Review Board (*Comitê de Ética em Pesquisa da Pontifícia Universidade Católica de Minas Gerais*) approved the use of the hospital database. Patients’ parents had signed an informed consent authorizing the use of exams for scientific purposes. The privacy of all subjects was protected.

From a population of 1234 children screened and treated by team of otolaryngologists, allergists, speech pathologists, physical therapists and orthodontists, between November, 2002 and September, 2014, at the Hospital das Clínicas da Universidade Federal de Minas Gerais, hospital, 28 individuals who had been submitted to multi-slice computed tomography (MSCT) scans, in addition to conventional ENT clinical and endoscopic examinations, were selected. All these children were diagnosed as obstructive sleep apnea (OSA) patients by polissonography and referred to a MSCT study to better clarify the levels of the upper airway obstruction. MSCT obtained for patients with syndromes, nasal septum deviation, craniofacial malformations and previous ENT surgical procedures were not included in the sample. After excluding three subjects, because of the poor quality of their exams, not adequate for research purposes, the total convenience sample was composed of 25 children (13 girls and 12 boys), 5–9 years old (mean = 6.56 years, median = 6 years).

The anatomical airway landmarks proposed in this investigation have not been used in previous publications. Therefore, the sample size was calculated based on the standard deviation of the analysis of the first consecutive 10 cases. At least 12 patients should be included in each ENT-diagnosis group (obstructed vs. non-obstructed). Obstruction should be located in at least one of the three anatomical sites (nasal cavity, nasopharynx and oropharynx), based on an alpha significance level of 0.05 and a beta level of 0.2 to achieve a power of 80% to detect a mean difference of 10% between the groups.

### ENT assessment

**Figure 1:**
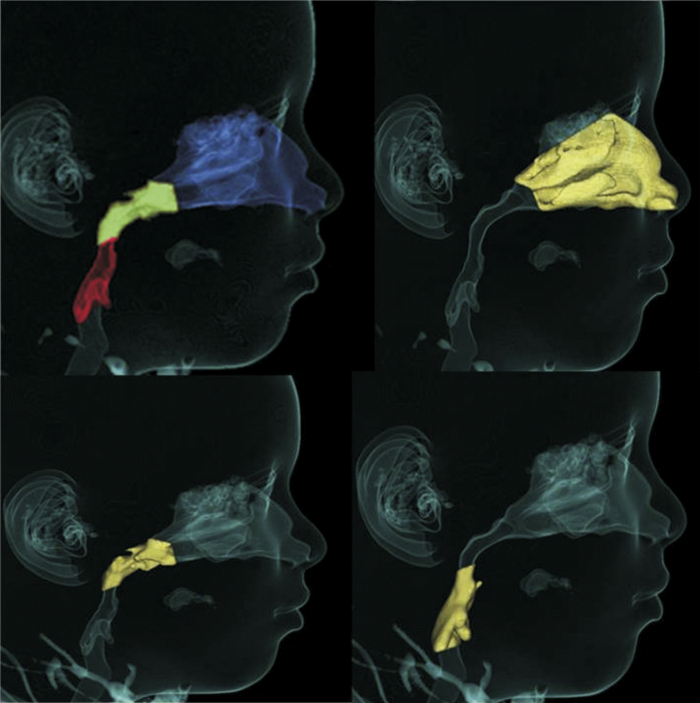
Airways measurement. A, nasal cavity; B, nasopharynx; C, oropharynx.

Based on the clinical and endoscopic ENT examinations performed by two of the authors at the first consultation, the obstruction of upper airway obstruction could either be related to the nasal cavity, nasopharynx or oropharynx (Fig. 1). The endoscopic examination was performed by an experienced otolaryngologist, using a flexible nasolaryngoscope, 3.2 mm (Machida ENT-30PIII), associated with an endocoupler. Topical spray anesthesia (2% lidocaine with epinephrine at a concentration of 1:20,000) was previously administered to reduce discomfort.

The nasal cavity was considered obstructed when the inferior and/or middle turbinates were hypertrophic according to the anterior rhinoscopy and the obstruction persisted after nasal decongestion with a topical vasoconstrictor. It was considered nasopharyngeal obstruction when adenoidal hyperplasia was present, occupying more than 75% of the choanal region.^16^, ^17^ Oropharynx obstruction was diagnosed on the basis of Brodsky and Koch Grade 3 or 4 palatine tonsil hyperplasia.^18^

Based on the ENT clinical diagnosis, children were grouped according to the findings of the three anatomical regions of the upper airways. The nasal cavity and nasopharynx groups were composed of 13 obstructive subjects and 12 non-obstructive subjects each. The oropharynx group included 12 obstructive subjects and 13 non-obstructive subjects. Obstructed and non-obstructed children were sex and age matched.

### Volumetric measurement of the upper airways

Nineteen children had a MSCT image of the airways taken on the day of the ENT clinical examination, while the other six children were submitted to CT scan within the first week of ENT clinical examination. The MSCTs were acquired on the same equipment (Somatom multislice scanner, 128 units, Siemens, Erlangen, Germany) with 100 kV and a 36 mA current time of 1.57 s tube voltage acquisition. The radiologist responsible for the selected patients’ exams was trained, calibrated and was blinded to the results of clinical and endoscopic examinations performed by otorhinolaryngologists. The children had been instructed not to breathe deeply, not to swallow, and not to move their head and tongue during the scanning, and they were positioned in a supine position. The collimation was 1 mm, and the slice thickness was 0.6 mm.

**Figure 2:**
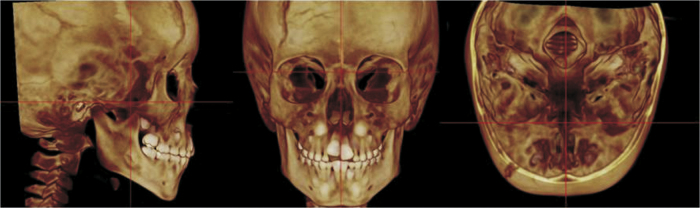
MSCT patient's orientation. A, sagittal view; B, coronal view; C, axial view.

To standardize the measurements and minimize the errors, the 3D constructed MSCT image was reoriented in the three spatial planes. In the frontal view (the coronal plane), the head was positioned with the line connecting the right and left fronto-zygomatic sutures parallel to the floor. In the right lateral view (the sagittal plane), the Frankfurt Horizontal (FH) plane was used as the reference plane, which was positioned parallel to the floor. FH was constructed from the right *porion*, located in the most laterosuperior point of the external auditory meatus, and the right *orbitale*. The superior view (the axial plane) was constructed through the *crista galli* and *basion*, and the line connecting the anatomical structure was aligned parallel to the mid-sagittal plane and perpendicular to the ground (Fig. 2).

**Figure 3:**
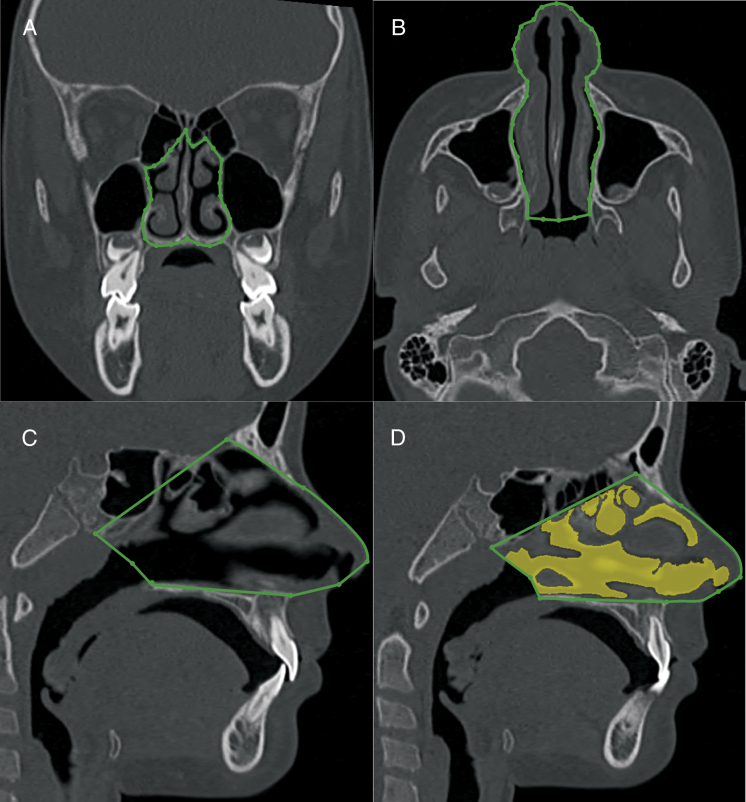
Nasal cavity volumetric MSCT measurement. A, coronal view, pyriform aperture boundaries. B, axial view, lateral walls of nasal cavity. C, sagittal view, anatomic landmarks described in Table 1. D, sagittal view of the nasal cavity volume.

**Figure 4:**
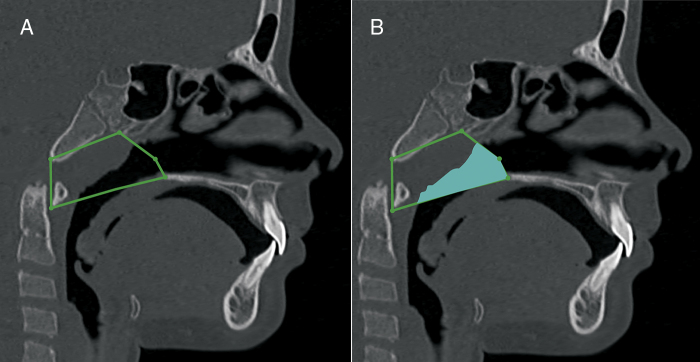
Nasopharynx volumetric measurement. A, sagittal view, anatomic landmarks described in Table 2. B, sagittal view of the nasopharynx volume.

**Figure 5:**
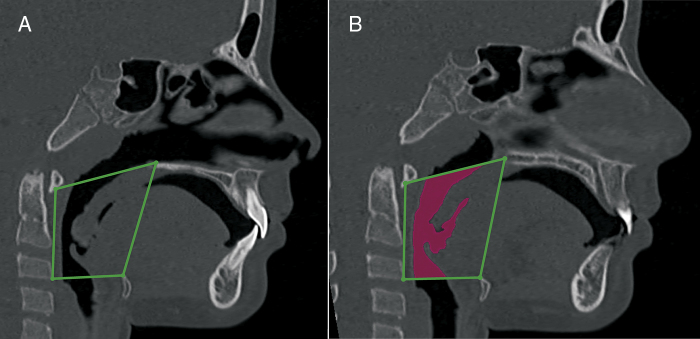
Oropharynx volumetric measurement. A, sagittal view, anatomic landmarks described in Table 3. B, a sagittal view of the oropharynx volume.

The volumetric construction and measurements were performed by an experienced radiologist with previously calibrated specific tools to calculate the volume of airways (Figure 3, Figure 4, Figure 5) (3D-mode airway/sinus Dolphin Imaging software, version 11.5, Chatsworth, CA, USA). The threshold value was set at 73,^9^ and the anatomical boundaries were established using the cranial technical points described in Table 1, Table 2, Table 3.

**Table 1:** Cranial landmarks used in the nasal cavity anatomic boundaries construction.

Cranial landmarks	Definition
Anterior nasal spine	Most anterior point of the floor of the nasal fossa, at the bottom of the nostril.
Posterior nasal spine	Most posterior point of the palatine bone and the floor of the nasal cavities.
Nasion	Point located in the center of the frontonasal suture
Nasal bone	The lowest point of nasal bone
Pterygomaxillary fissure	Lowest point of the pterygomaxillary fissure
Atlas	Most anterior inferior point of the Atlas vertebra
Vomer	Point located in the posterior portion of the vomer bone
Nostril	Anterior portion of the nostril opening
Nose	Anterior portion of the nose tip

**Table 2:** Cranial landmarks used in the nasopharynx anatomic boundaries construction.

Cranial landmarks	Definition
Posterior nasal spine	Most posterior point of the palatine bone and the floor of the nasal cavities
Vomer	Point located in the posterior portion of the vomer bone
Basion	Point located in the inferior and posterior limit of the anterior border of the foramen magnum
Pterygomaxillary fissure	Lowest point of the pterygomaxillary fossa
Atlas	Anterior inferior point of atlas vertebra

**Table 3:** Cranial landmarks used in the oropharynx anatomic boundaries construction.

Cranial landmarks	Definition
Posterior nasal spine	Most posterior point of the palatine bone and the floor of the nasal cavities.
Atlas	Anterior inferior point of atlas vertebra
Hyoid	Posterior inferior point of the hyoid bone
C3 vertebra	Superior-anterior point of the C3 vertebra

### Statistical methods

To determine errors in the landmark identification and measurements, 20 subjects were randomly selected, and a second investigator remeasured the identical scans. The first investigator remeasured fourteen subjects, after an interval of one month. The intraclass correlation coefficients (ICC) was calculated to assess the interexaminer and intraexaminer agreement. For the assessment of the differences between the obstructed and non-obstructed children, we used an independent sample *t*-test. The assumptions of normality within each group (Kolmogorov–Smirnov test) and homoscedasticity (Levene) were performed.

The significance level was set at *p* < 0.05. The data were analyzed using SPSS, version 20.0 (SPSS, Inc., Chicago, IL, USA).

## Results

The ICC was 0.99 for the interobserver nasal cavity and oropharynx evaluation and 0.97 for the interobserver nasopharynx evaluation. The ICC was 0.98 for the intraobserver nasal cavity evaluation and 0.98 for the nasopharynx and oropharynx intraobserver evaluation. Reproducibility of the method was considered nearly perfect.

**Figure 6:**
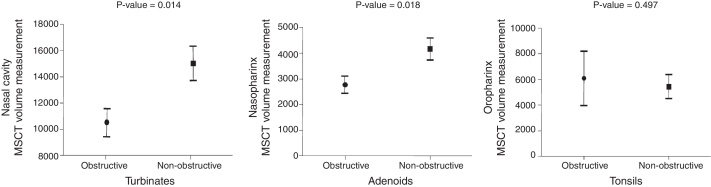
MSCT 3D volumetric measurement of A, nasal cavity; B, oropharynx; and C, oropharynx in obstructed and non-obstructed subjects according to ENT clinical and nasoendoscopy diagnosis.

Fig. 6 show histograms with the comparison between the MB subjects with ENT diagnoses of obstructive and non-obstructive airways in the three anatomical sites (nasal cavity, nasopharynx and oropharynx).

The MSCT-3D nasal cavity measurements showed a 30% volume reduction (*p* < 0.05) in the MB children with obstructive turbinates, in comparison with the MB children whose turbinates were considered within normal limits (10,564 vs. 15,073 mm^3^). Additionally, the nasopharynx volume measurement was associated with lymphatic tissue hyperplasia. The children whose adenoids where considered obstructive according to the ENT examination presented a statistically significant reduced nasopharynx volume (2757 vs. 4143 mm^3^) in comparison with the non-obstructive adenoid MB children (*p* < 0.05), which represents a 33% airway volume reduction. The MSCT measurements of the oropharynx volume were not associated with obstructive tonsils. The MB children with obstructive tonsils showed an oropharynx measurement of 6094 mm^3^, whereas the children without obstructive tonsils showed a 5453 mm^3^ measurement. The 11.5% reduction of the oropharynx airway volume of the MB children without obstructive tonsils was not statistically significant (*p* = 0.497).

## Discussion

Excellent intra- and interobserver agreement (ICC 0.97–0.99) was found in this investigation. This high reproducibility has also been described in previous studies with volumetric measurements of upper airways.^5^, ^6^, ^9^, ^14^ However, the validity of the volumetric 3D-CT scan measurements of the upper airways merits concern and should be better assessed.^6^, ^19^ The objective of this study was to evaluate whether the 3D measurements of the nasal cavity, nasopharynx and oropharynx are in agreement with the ENT gold standard clinical diagnosis. To increase the validity of the measurements, we selected the anatomical landmarks, rather than the reference lines and planes, used in the previous studies, which did not accurately portray the boundaries of each region of the upper airways.^8^, ^11^, ^15^, ^20^ We hypothesize that the adoption of the actual anatomy of the compartments of the upper airspace is an improvement in the measurement method.

It was found that the reduction of the volume of the nasal cavity and nasopharynx are associated with hypertrophied turbinates and enlarged adenoids, respectively. But, when the tonsils were enlarged, no agreement was observed in the reduction of the 3D oropharynx volume. The 3D oropharynx volumetric measurement of tonsils’ obstructed group and tonsils’ non-obstructed group was statistically similar. This result was unexpected because it appears contradictory that the oropharynx could be at least partially occupied by enlarged lymphoid tissue and its volume remains unchanged. This finding might be attributed to more posterior positioning of the tongue during the examination of the patient in a supine position during the MSCT scan.^4^, ^17^, ^21^ This posterior drop of the tongue within the oropharynx, which appeared in all of the subjects, might have masked the actual volume of that anatomical site in the MB children without tonsil enlargement. Therefore, a false-positive reduction of the airways might be seen in CT scans of patients in supine position. Our findings in regard the oropharynx must be different than former studies that used cone beam computed tomography (CBCT) in seated position. However, we understand that when children are in a supine position, they are placed in a condition closer to the sleeping position than if they were seated, which is used by most CBCT equipment. Another advantage of MSCT, in comparison with CBCT, is the acquisition time. MSCT is significantly faster (1.57 s), in contrast with that of CBCT (40 s), facilitating the examination of non-compliant young children and reducing the bias of respiratory movements that occurs in time-consuming examinations.^21^, ^22^

Several methodologies have been proposed for airway evaluation on lateral radiographs^1^, ^2^, ^10^ and CT images.^6^, ^8^, ^14^, ^23^ A revision of the previously published data on this topic shows that the anatomical definition of the airways has been extremely variable and not based on precise anatomical landmarks.^8^, ^10^, ^13^ In this study we used the actual anatomical landmarks, as defined in Table 1, Table 2, Table 3. Instead of using the anterior nasal spine as the anterior limit of the nasal cavity,^6^, ^15^, ^24^ we considered the full nose extension to the nostril opening for the volumetric measurements. Additionally, the upper limit of the nasal cavity was improved. In a previous study, the height of the nasal cavity was significantly under measured. To improve the validity of the nasopharynx measurements, the choana was considered the anterior wall. Previous studies did not evaluate the nasopharynx adequately because, in most cases, a significant portion of the nasal cavity was included in the nasopharynx.^6^, ^10^, ^20^

This is a pioneer study in the assessment of changes in the 3D volume of specific sites of the upper airways. Our measurement method and findings can contribute in future scientific investigations of 3D assessment of upper airways because it is based on actual anatomic landmarks and not convenient landmarks, which did not represent the real anatomic region. The evidence in the present investigation confirmed that the volumetric assessments of the upper airways by MSCT are quite reproducible. We added information showing that it is likely that turbinate hypertrophy and adenoid hyperplasia would be found in the cases in which the nasal cavity and nasopharynx volumes are reduced. The volume of the oropharynx from the MSCT measurements is not associated with obstruction by the palatine tonsils. Additional research is needed to elucidate the association between palatine tonsil obstruction and its effect on the airways in MB children in seated and as well supine position.

As a final statement, it is important to note that the indication of MSCT is not routine in the evaluation of mouth-breathing children and should be reserved for exceptional, but not so rare, cases.

## Conclusions

Based on the volumetric MSCT findings and the ENT diagnosis, the following conclusions could be inferred:

1) The nasal cavity volume was reduced in the patients in which hypertrophic turbinates were diagnosed.
2) The nasopharynx was reduced in the patients in which hyperplasia adenoids were diagnosed.
3) The oropharynx volume of mouth breathing children with obstructed tonsils was similar to that of non-obstructed mouth breathing children.

## Conflicts of interest

The authors declare no conflicts of interest.
